# Comparative thermodynamic studies on substrate and product binding of O-Acetylserine Sulfhydrylase reveals two different ligand recognition modes^†^

**DOI:** 10.1186/1471-2091-12-31

**Published:** 2011-06-02

**Authors:** Shrijita Banerjee, Mary K Ekka, Sangaralingam Kumaran

**Affiliations:** 1 Council of Scientific and Industrial Research, Institute of Microbial Technology, Sector 39-A, Chandigarh, 160036, India

**Keywords:** Ligand Binding, Enthalpy, Entropy, Fluorescence, Isothermal Titration Calorimetry

## Abstract

**Background:**

The importance of understanding the detailed mechanism of cysteine biosynthesis in bacteria is underscored by the fact that cysteine is the only sulfur donor for all cellular components containing reduced sulfur. O-acetylserine sulfhydrylase (OASS) catalyzes this crucial last step in the cysteine biosynthesis and has been recognized as an important gene for the survival and virulence of pathogenic bacteria. Structural and kinetic studies have contributed to the understanding of mechanistic aspects of OASS, but details of ligand recognition features of OASS are not available. In the absence of any detailed study on the energetics of ligand binding, we have studied the thermodynamics of OASS from *Salmonella typhimurium *(*St*OASS), *Haemophilus influenzae *(*Hi*OASS), and *Mycobacterium tuberculosis (MtOASS) *binding to their substrate O-acetylserine (OAS), substrate analogue (methionine), and product (cysteine).

**Results:**

Ligand binding properties of three OASS enzymes are studied under defined solution conditions. Both substrate and product binding is an exothermic reaction, but their thermodynamic signatures are very different. Cysteine binding to OASS shows that both enthalpy and entropy contribute significantly to the binding free energy at all temperatures (10-30°C) examined. The analyses of interaction between OASS with OAS (substrate) or methionine (substrate analogue) revealed a completely different mode of binding. Binding of both OAS and methionine to OASS is dominated by a favorable entropy change, with minor contribution from enthalpy change (ΔH_St-Met _= -1.5 ± 0.1 kJ/mol; TΔS_St-Met _= 8.2 kJ/mol) at 20°C. Our salt dependent ligand binding studies indicate that methionine binding affinity is more sensitive to [NaCl] as compared to cysteine affinity.

**Conclusions:**

We show that OASS from three different pathogenic bacteria bind substrate and product through two different mechanisms. Results indicate that predominantly entropy driven methionine binding is not mediated through classical hydrophobic binding, instead, may involve desolvation of the polar active site. We speculate that OASS in general, may exhibit two different binding mechanisms for recognizing substrates and products.

## Background

Cysteine biosynthesis in bacteria is a highly regulated process. Enzymes involved in cysteine biosynthesis function as molecular sensors [1-3]. The de novo biosynthesis of cysteine is catalyzed in two steps. In the first step, serine acetyltransferase (SAT) catalyzes the formation of OAS from acetylCoA and L-serine. In the second step, OAS is converted to cysteine by O-acetylserine sulfhydrylase (OASS) through elimination of acetate and addition of bisulfide [4-6]. Since cysteine is the only sulfur donor for all cellular components containing reduced sulfur, balanced activity of OASS is very important for growth and survival of the bacterium [7]. In addition to its role in protein structure, cysteine is the precursor for the biosynthesis of a variety of primary and secondary metabolites like glutathione, vitamin cofactors, anti-oxidants, etc [8,9]. The conservation of cysteine biosynthesis genes including OASS in many intracellular pathogens and up regulation of OASS during bacterial infection suggests that OASS is essential for the survival of these pathogens [10,11]. Loss of OASS has been shown to affect both the survival fitness of *Salmonella *under hostile conditions and virulence of the bacterium [12]. Thus, inhibition of OASS using specific inhibitors should result in reduced survival of bacteria inside the macrophage. Absence of OASS homologues in humans makes it an attractive antibacterial drug target. A recent study has exploited the protein-protein interaction properties of OASS and SAT to design small peptides that selectively bind to OASS with higher affinity and inhibit its activity [13].

Crystal structures of OASS from *S. typhimurium *in the presence and absence of ligands have been determined [14,15]. Three-dimensional structure of OASS from *H. influenzae *has been determined in complex with C-terminal peptide of serine acetyltransferase [16]. Structural studies show that OASS is a homo-dimer and it belongs to the *β*-family of PLP-dependent enzymes with one PLP/subunit buried within the protein. In the absence of bound ligand, the coenzyme pyridoxal 5'-phosphate (PLP) is bound via a Schiff base to the side chain of Lys41 [14]. Activity of OASS is regulated by its metabolites and other enzymes involved in cysteine metabolism. Spectroscopic properties of active site bound PLP has been exploited to investigate the ligand recognition principles of OASS [17]. Structural and biochemical studies have shown that OASS can bind both cysteine, its product, and methionine, substrate analogue of OAS [15,17-19]. Comparison of crystal structures of methionine bound *S. typhimurium *OASS (*StOASS-met) *and cysteine bound *E. histolytica *OASS (*EhOASS-cys) *indicates that both amino acids are found in the active site center with their α-amino group oriented towards C4A atom of PLP [15,18,19]. Therefore, it is expected that both cysteine and methionine may bind to the active site through similar mechanisms. Ligand binding to *S. typhimurium *(*StOASS*) active site causes conformational changes in the protein [15]. Although structures of complexes offer glimpses of ligand occupied active site of OASS, thermodynamic properties of protein-ligand interactions have not been studied. Catalytic mechanism of OASS has been studied in detail [20,21], and studies also show that OASS exhibits both substrate and product inhibition [6]. Further, biochemical properties of cysteine synthesis enzymes are influenced by binding of substrates, products, small ions, and other proteins [3,15,17,18]. Understanding the energetics of interactions of these small molecules with proteins would provide information on regulatory features such as binding modes for ligands. Quantitative characterization of energetics of protein-ligand interactions would provide additional information which is necessary to understand the details of ligand recognition features of an enzyme.

Studies on biochemical characterization of OASS have used mostly steady state as well as pre-steady state kinetic approaches to understand the catalytic mechanism. Physiological mechanism of an enzyme deduced only from kinetic studies is incomplete in the absence of information on ligand binding properties. Although structural studies have provided information about residues that mediate ligand binding, ligand recognition mechanisms are studied using thermodynamic and kinetic approaches [22]. Isothermal titration calorimetry is the most direct method for the determination of heats of binding (ΔH_bind_) and binding free energy (ΔG_bind_) upon protein ligand binding [23]. In the absence of any detailed study to understand the energetics of substrate and product interaction with OASS, we present here a detailed thermodynamic description of ligand binding by OASS from *S. typhimurium, M. tuberculosis*, and *H. influenzae*. We focused on studying the interaction of OASS with cysteine, O-acetyl serine (OAS), and methionine as a function of temperature and determined the relative contributions of enthalpy (ΔH) and entropy (ΔS) to the binding free energy. We observed a predominantly entropy driven binding for substrate and substrate analogue. We also examined these protein-ligand interactions as a function of salt concentrations and studied the salt dependency of OASS-ligand interactions. Substrate binding is more sensitive to salt concentrations than product binding. Our studies reveal that binding of substrate and product exhibit completely different thermodynamic signatures. Product binding is driven by both favorable enthalpic and entropic contributions whereas substrate binding is predominantly driven by entropic contribution.

## Results

### Characterization of ligand binding of OASS by fluorescence spectroscopy

**Figure 1:**
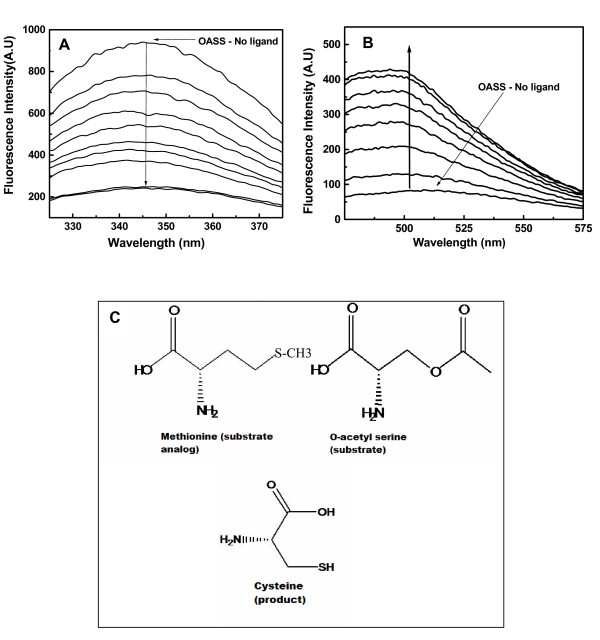
**Spectroscopic characterization of OASS and ligand binding**. (A) Fluorescence emission of OASS upon excitation at 295 nm and quenching of fluorescence due to ligand binding (down arrow indicates decrease in fluorescence upon addition of ligands). (B) Fluorescence emission of OASS upon excitation at 412 nm (PLP) and change in fluorescence intensity due to methionine binding (upward arrow indicates increase in PLP fluorescence upon addition of ligands). Fluorescence increase at 507 nm was used for determining the extent of binding. (C) Cartoon representation of substrate (OAS), substrate analogue (methionine), and product (cysteine) used in this study.

**Figure 2:**
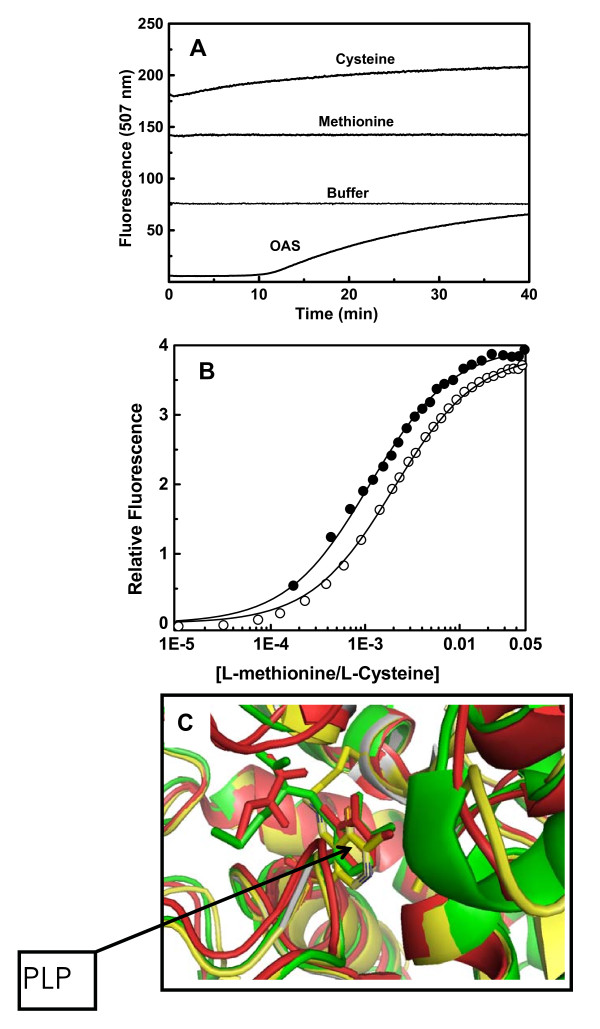
**Monitoring ligand binding by fluorescence spectroscopy**. (A) Time dependent PLP fluorescence of OASS (1 μM) after addition of buffer, OAS (4 μM), methionine (1.4 mM), and cysteine (1.3 mM) as indicated in the figure. (B) Fluorescence quenching titrations of the *St*OASS with cysteine (closed circle) and methionine (open circle); the data from both sets were fit to two identical site model (eq 1) independently. The solid line represents the best fit to data that yields K_cysteine _= 6.0 × 10^2 ^M^-1^; K_met _= 2.9 × 10^2 ^M^-1^; (C) OASS-cysteine (red) and OASS-met (Green) complexes superimposed to show the interaction of ligand in the active site. Methionine is covalently attached to the active site PLP through Schiff base.

The absorbance spectra of OASS from all three species (*H. influenza*, *S. typhimurium*, *M.tuberculosis*) showed two distinct absorption maxima, one around 280 nm and a second maximum at 412 nm indicative of the presence of internal aldimine [17]. Excitation of OASS at 292 nm yields a fluorescence emission 325-350 nm due to Trp emission and excitation at 412 nm gives another emission spectrum in the range 475-540 nm due to PLP fluorescence. Fluorescence properties of OASS were investigated previously and it was observed that excitation at 298 nm generates a large increase in fluorescence between 340-345 nm [24,25]. Similar to earlier observations, excitation at 292 nm yields an emission spectrum centered at 345 nm and fluorescence at 345 nm is quenched upon ligand binding (Figure 1A). We have examined the binding of O-acetyl serine (OAS), L-cysteine, L-methionine, L-serine, and L-isoleucine to OASS by monitoring the changes in fluorescence properties of the protein. Binding of cysteine or methionine to OASS changes both tryptophan and PLP fluorescence spectra (Figure 1A &1B). Ligand binding quenches tryptophan fluorescence observed at 345 nm (Figure 1A), but increases the PLP fluorescence at 507 nm as observed earlier [26]. It was observed that excitation of OASS at 290 nm leads to fluorescence at 345 nm as well as at 500 nm due to energy transfer from tryptophan to PLP. Therefore, we monitored the increase in the PLP fluorescence emission at 507 nm upon excitation at 412 nm for constructing binding isotherms and determining the equilibrium binding parameters. Addition of OAS quenches the PLP fluorescence initially, but fluorescence signal increases as a function of time indicating the existence of a slow kinetic process after initial quenching (Figure 2A). Detailed studies are necessary to understand the molecular origin of this slow kinetic process.

Binding of methionine and cysteine to OASS results in the formation of external aldimines [27]. Pre-steady state studies observed the slow disappearance of α-aminoacrylate indicating that α-aminoacrylate may be hydrolyzed in the absence of sulfide [27]. Therefore, it is possible that slow increase of PLP fluorescence may represent hydrolysis of α-aminoacrylate or yet other uncharacterized processes. Methionine has been used as substrate analogue in earlier studies to understand the reaction mechanism [15]. Methionine forms stable external aldimine upon binding to OASS and yields a stable signal change which could be used for studying the interaction quantitatively.

In each titration, the relative PLP fluorescence increases with the addition of more ligands reaching a plateau upon saturation of binding sites on OASS (Figure 1B). Since OASS is a homo-dimer, we fitted all the binding isotherms in this study to a two-site binding model (eq1). Fitting yields similar values for K_1,obs _and K_2,obs _for each binding site. Binding of cysteine to *St*OASS yields K_1,obs _and K_2,obs _= 6.0 ± 0.1 × 10^2 ^M^-1 ^(K_d _~ 1.7 mM). Binding of methionine to *St*OASS yields K_1,obs _and K_2,obs _= 2.9 ± 0.1 × 10^2 ^M^-1 ^(K_d _~ 3.4 mM) and binding parameters for *Hi*OASS and *Mt*OASS are shown in table 1. Equilibrium binding constants obtained for methionine binding from our studies are similar to equilibrium constants estimated for OAS binding using single-wavelength stopped flow studies (K_ext _= 0.18 mM^-1 ^~ 1.8 × 10^2 ^M^-1^; K_d _~ 5.6 mM) [25]. Therefore, we studied the binding of methionine and cysteine for understanding the ligand recognition mechanisms of OASS (Figure 1C). First, we tested the secondary structural properties of OASS in the presence of these ligands. We compared secondary structural contents of unliganded enzyme and ligand bound forms using circular dichroism method. Results of CD spectra for both cysteine and methionine bound OASS are similar to that of unliganded OASS spectrum (Additional file 1, Figure S1). Crystal structure of *St*OASS-methionine complex showed that active site of OASS is in the closed form as compared to the structure of unliganded enzyme [14,15]. Secondary structural contents of both closed and open forms are almost similar as observed from crystal structures of unliganded and complex forms. We have also performed titrations of L-serine and L-isoleucine under similar experimental conditions. In contrast to earlier observation, addition of L-serine does not change OASS fluorescence under our solution conditions [17,27]. We found that increasing the pH of buffer to 9.5 leads to changes in the OASS fluorescence upon addition of L-serine (Additional file 1, Figure S2). We monitored the fluorescence signal change for determining the binding constant. Analysis of L-serine binding isotherm yields a K_d _value of ~ 3.7 mM which is less than the reported K_d _value of 4.2 mM from earlier study [17]. This difference may arise from differences in solution conditions (Additional file 1, Figure S2 and S3). Next, we studied the binding of isoleucine to OASS. OASS has been shown to interact with the C-terminal tail of serine acetyltransferase (SAT) [3]. The last residue at the C-termini of SAT is the highly conserved isoleucine and replacing this isoleucine with alanine decreases the affinity of SAT to OASS significantly [3]. We tested the possibility that OASS may specifically recognize isoleucine when presented as amino acid. Although isoleucine is the most important residue in determining the affinity between OASS and SAT, it does not bind OASS when presented as amino acid.

**Table 1:** Thermodynamic parameters associated with binding of cysteine and methionine to OASS

	Cysteine			Methionine		
**Temperature**	**ΔG (kJ/mol)**	**ΔH (kJ/mol)**	**TdS (kJ/mol)**	**ΔG (kJ/mol)**	**ΔH (kJ/mol)**	**TdS (kJ/mol)**

*St*OASS binding						

10°C	-9.6 ± 0.4	-2.7 ± 0.1	6.9	-8.6 ± 0.8	-1.3 ± 0.1	7.3

15°C	-9.6 ± 0.3	-2.8 ± 0.1	6.8	-8.6 ± 0.4	-1.4 ± 0.1	7.2

20°C	-9.8 ± 0.1	-6.4 ± 0.1	3.4	-9.7 ± 0.5	-1.5 ± 0.1	8.2

25°C	-10.6 ± 0.1	-5.9 ± 0.1	4.7	-9.5 ± 0.5	-0.9 ± 0.1	8.6

30°C	-10.2 ± 0.2	-4.4 ± 0.2	5.8	-10.4 ± 0.8	-1.0 ± 0.1	9.4

35°C	-11.0 ± 0.2	-5.4 ± 0.3	5.6	-11.3 ± 1.3	-1.2 ± 0.1	10.1

*Hi*OASSbinding						

10°C	-8.3 ± 0.4	-4.6 ± 0.1	3.7	-7.2 ± 0.9	-0.2 ± 0.1	7.1

15°C	-8.7 ± 0.2	-4.6 ± 0.2	4.1	-7.2 ± 0.9	-0.2 ± 0.1	7.5

20°C	-9.5 ± 0.5	-4.4 ± 0.2	5.1	-8.2 ± 1.0	-0.2 ± 0.1	8.0

25°C	-9.5 ± 0.5	-5.4 ± 0.3	4.1	-7.6 ± 1.1	-0.8 ± 0.1	6.9

30°C	-11.0 ± 0.3	-4.5 ± 0.4	6.5	-8.4 ± 1.0	-0.7 ± 0.1	7.7

Titrations were performed in 20 mM Tris buffer (pH 8.0), 50 mM NaCl. Data were fit to a two independent sites binding model that yielded similar values of ΔG, ΔH, and -TΔS for each site.

### Determination of OASS-ligand interaction energetics by ITC

**Figure 3:**
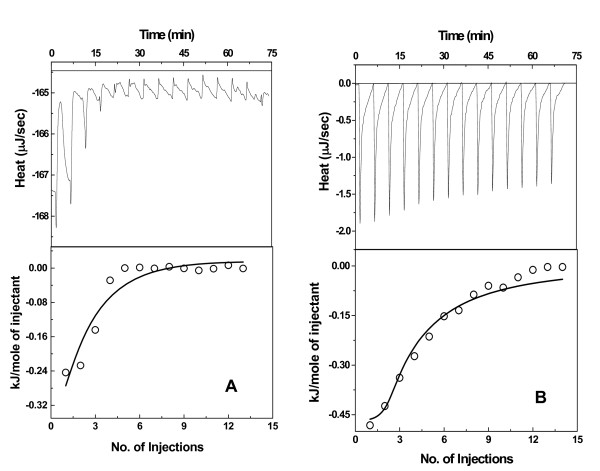
**ITC analysis of the interaction between substrate, OAS (O-acetylserine) and OASS**. Data is plotted as heat signal (μJ/sec) versus time (min) in the upper panel in each fig. Lower panel-integrated heat responses per injection from panel A plotted as normalized heat per mole of injectant. The solid line represents the best fit of the data to a two independent site binding model (eqn 2). (A) Titration of *St*OASS (4.5 μM) with OAS (5 mM) at 25°C. (B) Titration of *Hi*OASS (4.5 μM) with OAS (5 mM) at 25°C. Both titrations were performed in the same buffer (20 mM Tris, pH 8.0, 50 mM NaCl).

To the best of our knowledge, ITC has not been used to study ligand binding properties of OASS for quantifying the interaction parameters for OASS-ligand complex formation. The energetics of OASS binding to OAS, cysteine, methionine, serine, and isoleucine were examined. We performed isothermal titration calorimetry experiments at 25°C to probe the thermodynamics of OAS binding to OASS. Two titrations performed by injecting OAS to solution containing *St*OASS and *Hi*OASS are shown in Figure 3A and 3B. Heat signals were integrated, and the binding isotherm was analyzed using two independent site model. Two independent titrations show that binding of OAS is an exothermic reaction and binding isotherms can be fitted to the same model. The analysis of OAS binding to *St*OASS yielded an enthalpy value of 0.43 ± 0.2 kJ/M and an apparent binding constant of ~ 1.8 ± 0.6 × 10^3 ^M^-1 ^(ΔG = -17.6 kJ/M; K_d _= 0.56 mM). Similarly, analysis of interactions of other two OASS with OAS yielded enthalpy values of 0.45 ± 0.3 kJ/M (*Hi*OASS) and 0.53 ± 0.3 kJ/M (*Mt*OASS). The binding constants obtained for both *Hi*OASS and *Mt*OASS were similar (~ 1.3 ± 0.4 × 10^3 ^M^-1^; ΔG = -16.9 kJ/M; K_d _~ 0.77 mM for *Hi*OASS). In all three cases, the enthalpy contributes less than 3% to the total binding free energy. This is surprising because OAS is a small metabolite and has higher degrees of freedom in solution as compared to the bound form. Therefore, the entropy change upon complex formation is expected to be unfavorable for OAS-enzyme interaction. On the contrary, OAS interaction which includes its initial reaction and subsequent binding is predominantly driven by favorable entropy component in both cases.

**Figure 4:**
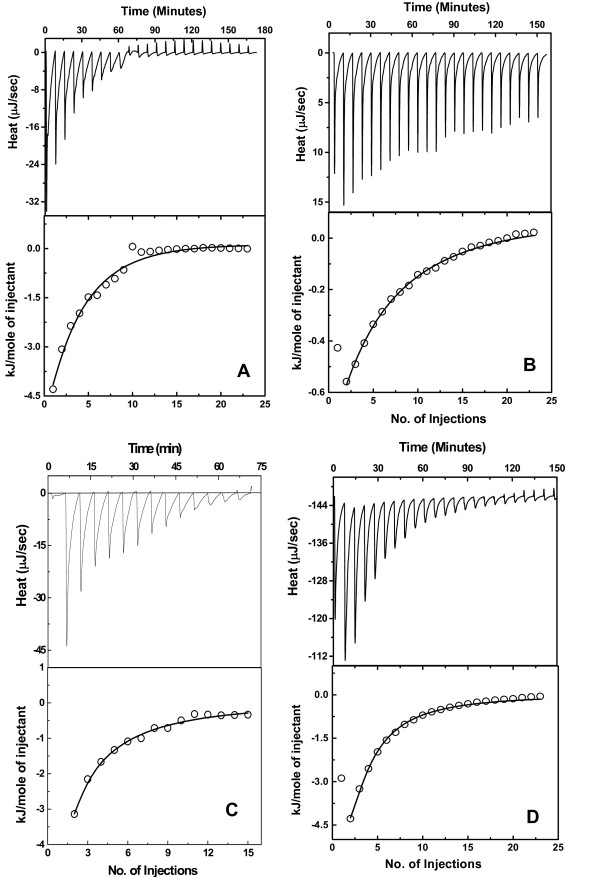
**ITC analysis of the interaction between OASS and cysteine**. Data is plotted as heat signal (μJ/sec) versus time (min) in the upper panel in each fig. Lower panel-Integrated heat responses per injection from panel A plotted as normalized heat per mole of injectant. The solid line represents the best fit of the data to a two independent site binding model (eqn 2). (A) Titration of *St*OASS (4.5 μM) with cysteine (350 mM) at 25°C. (B) Titration of *St*OASS (4.5 μM) with Methionine (350 mM) at 25°C. (C) Titration of *Mt*OASS (4.5 μM) with Cysteine (350 mM) at 25°C. (D) Titration of *Hi*OASS (4.5 μM) with cysteine (350 mM) at 25°C.

To compare the entropy driven binding of OAS with other ligands, we performed titrations using methionine (substrate analogue), and cysteine, the product of OASS. Titrations performed at 25°C indicate that binding of both cysteine and methionine to *St*OASS is exothermic (Figure 4A and 4B). Analysis of binding isotherms indicated that isotherms can be fitted to a two-sites binding model and fitting yielded similar values for each binding site (table 2). Interestingly, the binding reaction was observed to be entropically driven for methionine binding (TΔS_St-Met _= 8.2 kJ/M) with little contribution from enthalpy (ΔH_St-Met _= -1.5 ± 0.1 kJ/M) at 20°C (table 1). In contrast, cysteine binding is accompanied by both favorable enthalpic and entropic contributions (ΔH_St-Cys _= -6.4 ± 0.1 kJ/M; TΔS_St-Cys _= 3.4 kJ/M) at 20°C. In summary, our results indicate that both OAS and methionine binding is driven by favorable entropy whereas the cysteine binding is driven by both enthalpy and entropy.

**Table 2:** Fluorescence titration analysis of the salt-dependence of interaction between OASS and ligands

	Cysteine				Methionine			
**[NaCl] M**	**N**	**Qmax**	**K_obs _(M^-1^)**	**ΔG (kJ/mol)**	**N**	**Qmax**	**K_obs _(M^-1^)**	**ΔG (kJ/mol)**

*St*OASS binding								

0.02	2	3.8	6.0 ± 0.1 × 10^2^	-15.0 ± 0.1	2	3.8	2.9 ± 0.1 × 10^2^	-13.3 ± 0.1

0.05	2	4.6	5.6 ± 0.1 × 10^2^	-14.9 ± 0.1	-			

0.1	2	4.6	4.5 ± 0.1 × 10^2^	-14.3 ± 0.1	2	1.0	6.5 ± 0.1 × 10^1^	-10.0 ± 0.1

0.2	2	4.5	3.7 ± 0.1 × 10^2^	-13.9 ± 0.1	2	1.4	5.5 ± 0.1 × 10^1^	-9.4 ± 0.1

0.5	2	4.7	2.6 ± 0.1 × 10^2^	-13.0 ± 0.1	2	0.5	3.5 ± 0.1 × 10^1^	-8.7 ± 0.1

*Hi*OASS binding								

0.02	2	3.9	6.6 ± 0.1 × 10^2^	-15.2 ± 0.1	-	3.0	1.5 ± 0.1 × 10^2^	-11.8 ± 0.2

0.05	2	5.7	5.6 ± 0.1 × 10^2^	-15.5 ± 0.1	-			

0.1	2	5.8	4.5 ± 0.1 × 10^1^	-9.0 ± 0.1	2	0.5	5.8 ± 0.1 × 10^1^	-9.5 ± 0.5

0.2	2	5.8	3.1 ± 0.1 × 10^1^	-8.0 ± 0.1	2	0.2	3.4 ± 0.1 × 10^1^	-8.1 ± 0.5

0.5	2	3.1	3.2 ± 0.1 × 10^1^	-8.0 ± 0.1	2	0.2	2.3 ± 0.1 × 10^1^	-7.3 ± 0.6

Titrations were performed in 20 mM Tris buffer (pH 8.0), and indicated [NaCl]. Data were fit to a two independent sites binding model that yielded similar values of *K*_obs _for each site.

### Temperature dependence of cysteine and methionine binding to StOASS

To study the differential recognition of substrate and product by OASS, binding reaction was studied as a function of temperature (10-37°C). Titration of OAS at different temperatures and comparison and analysis of binding isotherms was difficult. OAS is an activated molecule, and its stability is temperature dependent. Although titration of OAS at 15°C provides binding isotherm, titrations at 30°C and 35°C show low signal to noise and are difficult to analyze (data not shown). Repeated experiments did not yield analyzable quality isotherms. Therefore, we examined the binding of methionine and cysteine as a function of temperature.

**Figure 5:**
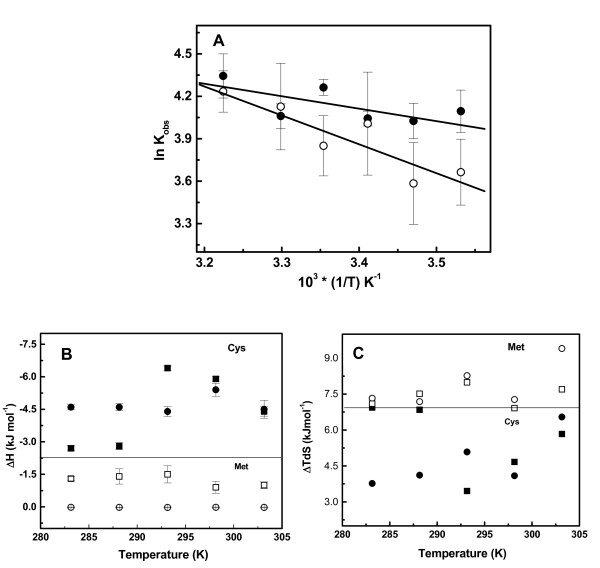
**Temperature dependence of thermodynamic parameters determined from ITC experiments**. (A) van't Hoff plot-temperature dependence of binding constants for cysteine and methionine binding to OASS; (closed circle) *St*OASS-methionine; (open circle) *St*OASS-cysteine; (B) Temperature dependence of binding enthalpy; (closed square) *St*OASS-cysteine; (closed circle) *Hi*OASS-cysteine; (open square) *St*OASS-methionine; (open circle-crossed) *Hi*OASS-methionine; horizontal line separates enthalpy for cysteine (Cys) binding from enthalpy contribution for methionine (Met) binding; (C) Temperature dependence of entropy; (open square) *St*OASS-methionine; (open circle) *Hi*OASS-methionine; (closed square) *St*OASS-cysteine; (closed circle) *Hi*OASS-cysteine; horizontal line separates net entropy change for cysteine (Cys) binding from entropic contribution for methionine (Met) binding;

The binding isotherms obtained at different temperatures were all exothermic and binding was accompanied with significant contributions from enthalpy and entropy. All binding isotherms were fit using a two-independent binding sites model. Binding enthalpy increases as temperature is raised and dependence of enthalpy on temperature yields a net heat capacity change (ΔC_p_= -130 JK^-1^M^-1^) for *St*OASS-cysteine complex formation. To investigate the predominantly entropy driven binding of methionine to OASS, we studied the *St*OASS-methionine interaction as a function of temperature to examine whether entropy remains as the major driving force at different temperatures (10-37°C). Although the methionine binding examined at all temperatures exhibited exothermic reaction similar to cysteine binding, binding at each temperature is accompanied with relatively large favorable entropic changes (table 1). Methionine binding reaction to *St*OASS shows that net enthalpic contribution is only ~ 10% of the total binding free energy (ΔH_St-Met _= -1.0 ± 0.2 kJ/M; ΔG_St-Met _= -10.4 kJ/M). In contrast, both enthalpic and entropic factors significantly contribute to cysteine binding. It is possible that linked process such as protonation or deprotonation can contribute to these differences. Dependence of observed enthalpy on ionization enthalpy of the buffer is the test for ligand binding coupled to proton exchange. To detect the potential contribution of protonation changes to the enthalpy of binding, we performed the titration of methionine to *St*OASS at pH 8.0 in HEPES buffer which has different ionization enthalpy (25 mM HEPES, 50 mM NaCl, at 25°C). Binding of methionine in HEPES buffer yields similar enthalpic contribution (ΔH_St-Met _= -0.4 ± 0.1 kJ/M) suggesting that proton exchange does not contribute to the observed methionine binding enthalpy. Similarly, cysteine-*St*OASS interaction was also examined at 25°C under similar conditions (25 mM HEPES, pH 8.0, 50 mM NaCl, 25°C). Analysis of cysteine binding isotherm yielded an enthalpic value of ~-4.0 ± 0.2 kJ/M (ΔH_St-Cys _= -3.9 kJ/M). Therefore, the higher enthalpic contribution of cysteine binding does not result from linked proton exchange equilibria, but it is the molar enthalpy of cysteine-OASS interaction. Although both cysteine and methionine bind to the same active site and have been shown to form external aldimine, thermodynamic parameters are qualitatively very different. Temperature dependence of binding constants show that van't Hoff plot is linear and cysteine binding shows more temperature dependency than methionine binding (Figure 5A). Temperature dependence of free energy change, enthalpy and entropy are plotted as a function of temperature in Figure 5B &5C, and dependency of binding constants are shown (Additional file 1, Figure S4). Temperature dependent enthalpy change of cysteine-*St*OASS interaction is not linear. However, both temperature dependent plots of enthalpy and entropy (Figure 5B &5C) show that methionine and cysteine binding parameters show clear dichotomy.

### Ionic dependence of ligand binding to OASS

**Figure 6:**
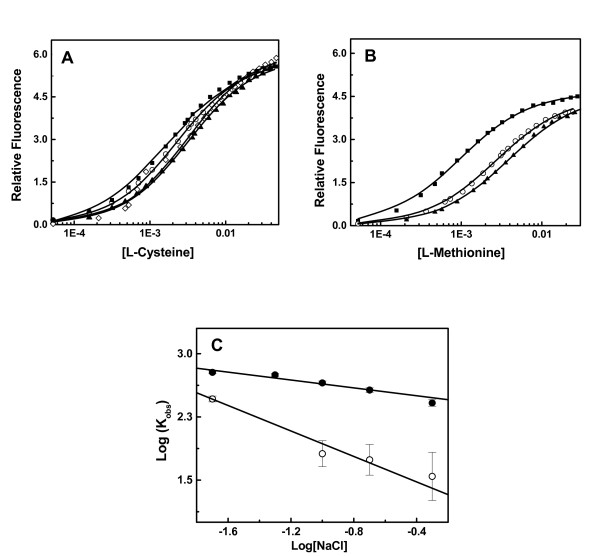
**Dependence of ligand-OASS interactions on ionic strength-Fluorescence quenching titrations of ligands in the binding buffer with indicated [NaCl]**. Solid line represents the fit derived from two identical site model; (A) Titrations of Cysteine with *St*OASS at indicated [NaCl] in the binding buffer; (closed square) 50 mM; (open circle) 100 mM; (open diamond) 200 mM; (closed triangle) 500 mM; (B) Titrations of methionine with *St*OASS at indicated [NaCl] in the binding buffer; (closed square) 100 mM; (open circle) 200 mM; (closed triangle) 500 mM; (C) Salt dependence of cysteine (closed circle) and methionine (open circle) binding constants. δlog(K_obs_)/δlog[NaCl] ~ 0.8 for methionine binding.

We have examined the interaction of methionine and cysteine with OASS under different solution conditions. Predominantly entropy driven methionine binding suggests that substrate binding may follow classical hydrophobic interactions with minimal contributions from other forces. On contrary, analyses of structure of OASS-methionine complex shows a number of polar interactions between the ligand's charged as well as polar groups and protein side chains suggesting the role of non-hydrophobic forces involved in methionine binding [15]. To understand the dependency of cysteine and methionine binding on electrolyte concentrations, we screened the complex formation in buffers with different ionic strength. Both the methionine and cysteine binding were examined by monitoring the binding processes as a function of [NaCl] (Figure 6A &6B). Binding of cysteine to *St*OASS showed that binding constants are not very sensitive to [NaCl] (dlogK/dlog [NaCl] ~ -0.21 ± 0.1) over the range of [NaCl] examined (0.02-0.5 M) (table 2). Interestingly, binding of methionine is more [NaCl] dependent than cysteine binding. Binding constant decreases as [NaCl] increased with dlogK/dlog [NaCl] value of ~ -0.8 ± 0.1, which indicates that methionine binding to *St*OASS is accompanied by the release of one anion or cation (Figure 6C). Comparison of binding constants of cysteine-*St*OASS and methionine-*St*OASS complex formation indicates that cysteine has at least 10 fold higher affinity for *St*OASS than methionine at higher [NaCl] (0.2-0.5 M).

### Determination of thermodynamic parameters for MtOASS and HiOASS interaction with ligands

We have examined the ligand binding properties of OASS from *Mt*OASS and *Hi*OASS to test whether the two different ligand recognition mechanisms observed in this study may be a common feature of bacterial OASS. Both enzymes were cloned and purified as described in materials and methods. Our OAS binding studies have shown that all three enzymes recognize their substrate through entropy dominated mechanism. Both methionine and cysteine were titrated versus respective OASS under similar conditions. Two independent titrations for each ligand were performed and binding isotherms were fit to two independent binding site model. Representative titrations of *Mt*OASS and *Hi*OASS binding to cysteine or methionine are shown in Figure 4C &4D. Binding of both cysteine and methionine to both *Mt*OASS and *Hi*OASS exhibit features which are very similar to features of *St*OASS interactions. Binding of both OAS and methionine are predominantly driven by entropy whereas cysteine binding is driven by both enthalpy and entropy. Analyses of binding isotherms yielded the respective thermodynamic parameters and are shown (table 1). The enthalpic contributions for methionine binding to *Mt*OASS and *Hi*OASS are 5% and 8% respectively. Our results presented here clearly show that both substrate and methionine binding to bacterial OASS, in general, may be entropy driven processes.

Since *St*OASS-ligand interactions showed two distinct binding modes for methionine and cysteine at all temperatures examined, we performed temperature dependent ITC experiments for *Hi*OASS-ligand interactions. Predominantly entropic driven behavior was observed for both *St*OASS and *Hi*OASS binding to methionine at all temperatures examined. However, specific differences are observed. On average, cysteine binds with higher affinity to *St*OASS than binding to *Hi*OASS (table 1). In contrast to temperature dependent enthalpy change in *St*OASS-cysteine interactions, *Hi*OASS-cysteine interaction showed relatively less temperature dependence as compared to *St*OASS-cysteine interaction (table 1). Hence the heat capacity changes (ΔC_p_= -130 JK^-1^M^-1^) for *St*OASS-cysteine complex formation is higher than heat capacity changes observed for (ΔC_p_= -12 JK^-1^M^-1^) *Hi*OASS-cysteine complex formation. In contrast, the affinity of cysteine for *Hi*OASS increases over 10 fold with increasing temperature. Increase in the binding affinity in the case of *Hi*OASS results from the temperature independent enthalpic contribution as well as increased entropic contribution at higher temperatures (table 1).

## Discussion

Prediction of thermodynamic parameters of protein-ligand interactions essentially requires studying the energetics of a large number of protein-ligand interactions in solution in addition to resolving the structures of those protein-ligand complexes. Studies on the ligand recognition features such as multiple binding modes provide information on the dynamic nature of an enzyme and essentially required to understand the variable effects of these ligands on enzyme function. Although structural and kinetic studies have enhanced our understanding of catalytic mechanism of OASS, ligand recognition mechanisms are not studied. In the present study, for the first time, we used ITC approaches to determine the thermodynamic parameters associated with the binding of OAS, methionine, and cysteine to OASS from *S. typhimurium*, *M. tuberculosis*, and *H. influenzae*. Comparison of detailed binding thermodynamics of three homologous enzymes involved in cysteine biosynthesis with these ligands show that bacterial OASS, in general, may recognize their substrate and product through two different binding mechanisms. In addition, two alternative binding modes for substrate and product binding were observed at all temperatures examined.

Our thermodynamic analyses indicate that binding of OASS to its ligands is accompanied by negative enthalpy change. It is known that formation of hydrogen bonds, hydrophobic, and ionic interactions between protein and ligand could contribute to the observed negative enthalpy change [28]. The favorable entropic contribution could result from desolvation of either ligand/protein binding sites upon interaction or from any conformational changes associated with the binding [29,30]. Bound OAS, cysteine, and methionine mediate hydrogen bonds and hydrophobic interactions with the active site residues of OASS. For example, *St*OASS-methionine structure shows that carboxylate group of methionine interacts with the side chains of THR 68, ASN 69, and GLN 144 [4]. Similarly, both α-amino group and carboxylate of cysteine also make several hydrogen bonds with side chain as well as main chain atoms of active site residues [15,18]. Based on these structural studies, one may expect that binding of either cysteine or methionine to OASS may proceed through similar mechanism in which both enthalpy and entropy would make significant contributions to the binding free energy. But predominantly entropy driven thermodynamic signatures of substrate and methionine binding suggest that OASS employs entropy dominated mechanisms for recognizing its substrates. Structural studies showed that methionine binding to *St*OASS induce global conformational changes that lead to closure of the active site [15]. Cysteine binding is also known to induce structural change which is similar to closed form observed for methionine binding. Since the structure of *Hi*OASS-methionine and *Mt*OASS-methionine complex are not available, it is difficult to correlate the structural changes observed in *St*OASS-methionine bound complex to entropy mediated binding mode observed in our study. Further, lack of structural information on cysteine-OASS complex for *St*OASS-cysteine and *Hi*OASS-cysteine prevents extrapolation of binding parameters to possible structural changes associated with methionine and cysteine binding.

Predominantly entropy driven binding has been assumed to be driven by classical hydrophobic effect. But cysteine binding to OASS has significant contribution from enthalpy. The hydrophobicity of methionine is slightly higher than that of cysteine [31]. Binding of the OAS also is driven by favorable entropy suggesting that both methionine and OAS are recognized in a similar manner. Methionine and OAS are structurally more similar and contain an additional carbon group which is a methyl in methionine and acetate group in OAS following the electronegative atom (sulfur in methionine and oxygen in the OAS). Therefore, it is conceivable that differential thermodynamic signatures observed for the binding of OAS/methionine and cysteine is due to the structural differences between substrate and product in general. Detailed thermodynamics of ligand binding properties of OASS enzymes from *S. typhimurium, M. tuberculosis*, and *H. influenzae *exhibited qualitatively very different thermodynamic signatures for substrate and product binding. To explain the entropically driven binding, methionine binding was studied as a function of [NaCl]. Our results do not favor the hypothesis that methionine binding is mediated through classical hydrophobic interactions because the binding affinity of methionine shows higher dependency on [NaCl] than cysteine affinity (table 2).

Entropy driven interactions are shown to result from higher desolvation of the binding interface [30]. Ligand binding induced conformational changes may also be responsible for such behavior if binding induces more disorder to the complex. Binding of methionine is known to change the protein conformation from open to close form, a relative more ordered form [15]. In general, conformational transitions that lead to more disordered form are responsible for favorable entropic contribution. Further, global conformational changes induced by binding would have resulted in non-linear (curved) temperature dependent enthalpy change [32]. The contribution of small conformational alterations, however, cannot be completely ruled out. Therefore, a major fraction of favorable entropy may result from other post-binding related process such as desolvation, protonation, etc. Our results presented here rule out the contribution of proton exchange suggesting that other processes like desolvation may contribute to the observed entropy change. Structures of *StOASS *complexed with methionine and *Eh*OASS complexed with cysteine show a number of water molecules present in the active site and few of them mediate interactions between ligands and proteins [15,18]. Since methionine and OAS have an extra methyl or acetate group, occupation of active site by these bulky ligands may lead to release of more number of non-specifically bound water molecules. Small changes in the structure of ligands (extra methyl group) are proposed to induce considerable changes in the binding [33]. Active site desolvation has been shown to play a role in enzyme catalysis [30,34]. The positive effect of substrate induced desolvation on enzyme reactivity has been recognized as an important phenomenon [34]. However, further experimental studies are needed to validate active site desolvation and test its role in OASS activity.

Enzymes involved in de novo cysteine biosynthesis have evolved with multiple regulatory strategies due to the need for tight control over physiological cysteine levels [1,3,35]. Therefore physiological activity of OASS is expected to be strictly controlled by temporal flux of metabolites. Equilibrium binding studies on interactions of metabolites with enzymes provide information on binding modes and energetics of interactions which are necessary to understand the regulatory mechanisms of enzymes. OASS has been shown to form non-covalent complexes with both substrate and product [15,18,23]. Although product inhibition of OASS has not been shown as one of the regulatory inhibition mechanisms, comparable affinity of cysteine for OASS suggest the possibility of product inhibition. At present, cysteine biosynthesis is known to be regulated by two mechanisms [3,35]. In the first mechanism, interaction of serine acetyltransferase (SAT) with OASS inhibits cysteine synthesis. The second mechanism operates through feed back inhibition in which cysteine, the product of OASS, inhibits SAT activity and regulates the availability of OAS which is the substrate for OASS. Intracellular cysteine levels are tightly controlled and increase in cysteine levels are known to increase oxidative DNA damage [36]. Considering the key position of OASS in the cysteine biosynthesis pathway, additional regulatory steps other than the two already known mechanisms may exist for fine tuning the cysteine biosynthesis. Alternative binding modes captured in this study may reflect the dynamic nature of this enzyme in recognizing various ligands. Therefore, identifying new molecular features of this enzyme is important to explore yet undiscovered regulatory mechanisms by which OASS may control cysteine flux. Further studies addressing the molecular origin of ligand recognition mechanisms are necessary to underscore the importance of alternative binding modes of OASS.

## Conclusions

For the first time, we have quantified the thermodynamic parameters for OASS binding to its ligands. We found that OASS recognizes its substrate and product through two different mechanisms. Our results presented in this study suggest that substrate binding is predominantly driven by favorable entropic change which may be caused by either active site desolvation or subtle conformational alterations upon binding. We have examined OASS from three different pathogenic bacteria and all three of them show alternative binding modes for substrate and product. Systematic studies on enzyme-ligand interaction studies may reveal more enzymes that may have evolved to recognize substrate and product through different binding modes. Further studies aimed at dissecting molecular origin and structural determinants of substrate and product binding modes are necessary to understand molecular plasticity of enzymes in ligand recognition.

## Methods

### Reagents and Buffer

All chemicals used were reagent grade and buffers were made with double distilled-water. O-acetylserine, L-cysteine, L-serine, L-methionine, and L-isoleucine were obtained from Sigma.

### Expression and purification of OASS

OASS (CysK) genes from *S. typhimurium, M. tuberculosis (H37Ra) *and *H. influenzae *were cloned into pET28a(+) vector and vectors containing OASS were transformed in to BL21(DE3) strain for expression. Protein expression was induced using 1 mM IPTG and the induction was carried out at 20°C for 16 hours with shaking at 220 rpm. Cells were harvested by centrifugation at 4,000 rpm and lysed by sonication. The soluble fraction containing OASS was recovered by centrifugation at 12,000 rpm. The N-terminally His tagged protein was purified using Ni-NTA affinity chromatography followed by gel filtration chromatography on Sepharose-S200 column. The purity of OASS was monitored on SDS PAGE followed by Coomassie brilliant blue R-250 staining. The purity of proteins were found to be > 98%. The purified enzymes were active (K_m,OAS _~ 0.9-1.4 mM; *k_cat _*~ 142, ~109 mM/min/μg for *StOASS *and *HiOASS *respectively) and fitting of *StOASS *of steady state kinetic data is shown (Additional file 2, Figure S1). We have determined the PLP/active-site ratio optically and data is submitted in the supplementary (Additional file 2, Figure S2). Spectroscopic determination of A280/412 absorbance ratio for all OASS enzymes used in this study yielded values ~ 2.6, as expected for 1:1 ratio of PLP bound to one active site [22]. Also we performed activity assays at different enzyme concentrations to check the linearity between enzyme concentration and activity (Additional file 2, Figure S3).

### Fluorescence Measurements

Titrations of ligands with *St*OASS were examined by monitoring changes in the active site pyridoxal 5'phosphate (PLP) fluorescence of OASS using a Varian spectrofluorometer. The excitation and emission wavelengths for monitoring PLP signal change upon ligand binding were 412 nm and 507 nm respectively. All experiments were done at 23.0 ± 1°C. Slit widths were set to 5 nm for all experiments and PMT voltage was adjusted to get maximum signal for a given protein concentration. Initial readings of both the sample, F_samp,0 _and buffer, F_buf,0 _were taken, with F_0 _= F_samp,0_-F_buf,0 _defined as the initial fluorescence of the sample. The sample cuvette was then titrated with aliquots of ligands and mixed, equilibrated for 2-5 minutes before measurement. Data points from five such measurements were averaged to obtain F_ave,i_. The relative fluorescence quenching upon ligand binding is defined as Q_obs,i _= (F_0_-F_obs,i_)/F_0_. All measurements were corrected for dilution, and inner filter effects.

Binding of ligands to OASS was analyzed to obtain the equilibrium binding constant, K_obs _= [PL]/[P]*[L], using a single or two independent site binding models (eqn 1)(1)

Where n, number of binding sites, *Q_obs_*, observed fluorescence quenching and *Q_max_*, the maximum fluorescence quenching at saturation.

### Calorimetric Measurements

Isothermal titration calorimetry (ITC) experiments were performed using a Nano-ITC (TA-Instruments). OASS was dialyzed at 25°C in 20 mM Tris buffer (pH 8.0) and 50 mM NaCl. Buffer solutions were degassed at room temperature prior to use. Briefly, injections of 10 μL of either methionine or cysteine dissolved in the dialysis buffer and added using a computer-controlled 250 μL microsyringe at an interval of 5-6 minutes into the sample solution containing either *St*OASS or *Hi*OASS. The heat of dilution for each injection was determined using buffer in the cell. Data obtained from the titration of ligands with OASS were analyzed using either a two-sites binding model (eqn 2)(2)

Q_i_^tot ^is total heat after the i^th ^injection, V_0 _is the volume of calorimetric cell, *K*_1 _and *K*_2 _are the observed equilibrium constants for each site, and ΔH_1 _and ΔH_2 _are the corresponding enthalpy changes. Estimates of *K*_obs _and ΔH were obtained by fitting the experimental data to the model and the best-fit parameters were selected based on the lowest Chi-squared values.

## Abbreviations

*St*OASS: *Salmonella typhimurium *O-acetylserine sulfhydrylase; *Hi*OASS: *Haemophilus influenzae *O-acetylserine sulfhydrylase; *MtOASS*: *Mycobacterium tuberculosis *O-acetylserine sulfhydrylase; *EhOASS*: *Entamoeba histolytica *O-acetylserine sulfhydrylase; Tris: tris(hydroxymethyl)aminomethane; ITC: Isothermal Titration Calorimetry; PLP: pyridoxal 5' phosphate.

## Authors' contributions

SK designed the concepts and experiments of the study. SK, SB cloned the genes. SB expressed, purified and performed all spectroscopic and thermodynamic experiments. MKE performed kinetic experiments. All authors have approved the final manuscript.

## Supplementary Material

Additional file 1 **Spectroscopic characterization of secondary structural contents of OASS and ligand binding**. Analyses of secondary structural contents of OASS using CD spectroscopy. L-serine binding studied by fluorescence spectroscopy. Click here for file

Additional file 2 **Spectroscopic and steady-state kinetic characterization of OASS**. Catalytic competency of enzyme was analyzed by spectroscopic and kinetic experiments. Click here for file
