# Supplementary material for: Comparative thermodynamic studies on substrate and product binding of O-Acetylserine Sulfhydrylase reveals two different ligand recognition modes†
Source: BMC Biochem. 2011 Jun 2;12:31. doi: 10.1186/1471-2091-12-31 (PMC3141655; doi:10.1186/1471-2091-12-31)
Supplement: Additional file 1 — Spectroscopic characterization of secondary structural contents of OASS and ligand binding. Analyses of secondary structural contents of OASS using CD spectroscopy. L-serine binding studied by fluorescence spectroscopy. [file 1471-2091-12-31-S1.PDF]

Figure S1

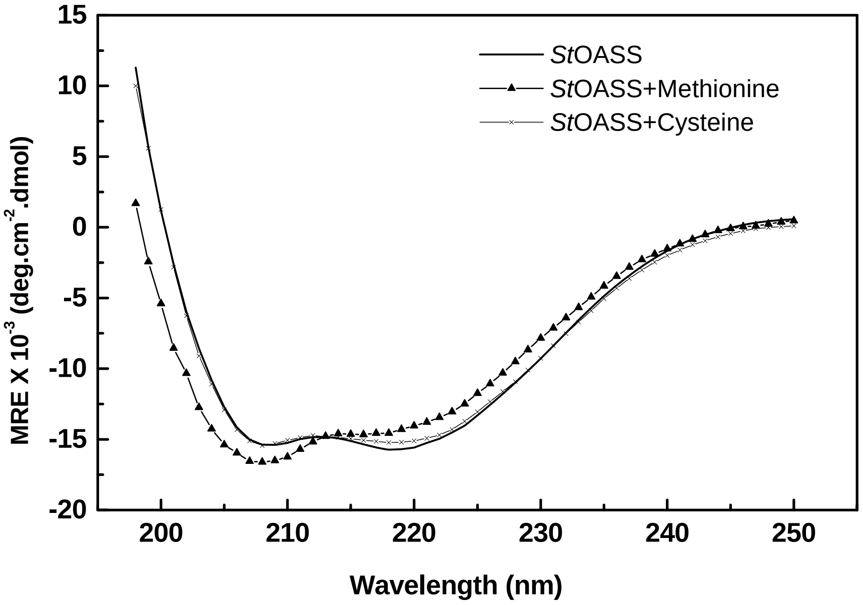

Circular dichroism spectroscopy measurements of *St*OASS and *St*OASS-ligand complexes at 25º C. Spectra for both cysteine and methionine were subtracted from protein-ligand complex spectra before plotting. Analysis of crystal structure of *St*OASS estimates ~34-36% α-helix and ~18 % β-sheet present. Secondary structural anlaysis of spectra show that both unliganded OASS and cysteine bound forms have almost similar secondary structural content (α-helix ~ 29-31%, and β-sheet ~ 20%). Methionine bound form shows slightly lower (α-helix content (~29 %). Due to the large uncertainty associated with the quantitative estimation of secondary structure contents from CD spectra, predicted changes may not reflect actual changes. Ellipticity at 222 nm is slightly less for methionine-OASS complex suggesting that α-helix content may be low for this complex.

CD measurements were carried out with a JASCO-810 spectropolarimeter (Jasco, Tokyo, Japan) equipped with a Peltier type temperature controller (PTC-348W). Far-UV spectra were obtained in a quartz cuvette with a 1mm light path-length and each spectrum obtained was an average of 10 scans. The ellipticity of protein CD spectra is reported as mean residue ellipticity (MRE) in deg/cm^2/dmol units. Deconvolution of far UV-CD spectra was performed using software provided by manufacturer (Jwsse32).

Figure S2

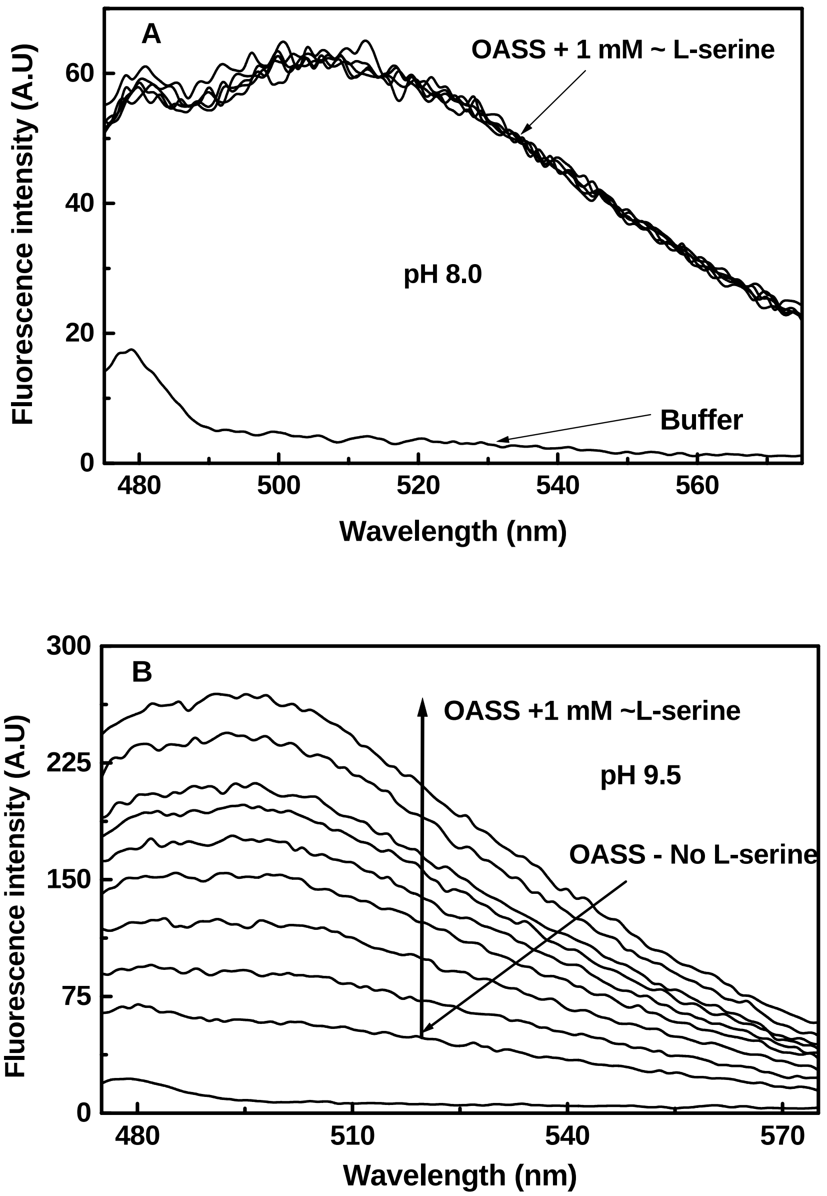

pH dependent binding of L-serine to OASS. A. Addition of L-serine does not change PLP fluorescence of OASS at pH 8.0 (20 mM Tris, 20 mM NaCl). B. Addition of L-serine increases the PLP fluorescence at pH 9.5 (20 mM Tris, 20 mM NaCl). Titrations were performed with similar concentrations of L-serine after each addition and titrations were continued till the final L-serine concentration is equal to ~ 1 mM.

Figure S3

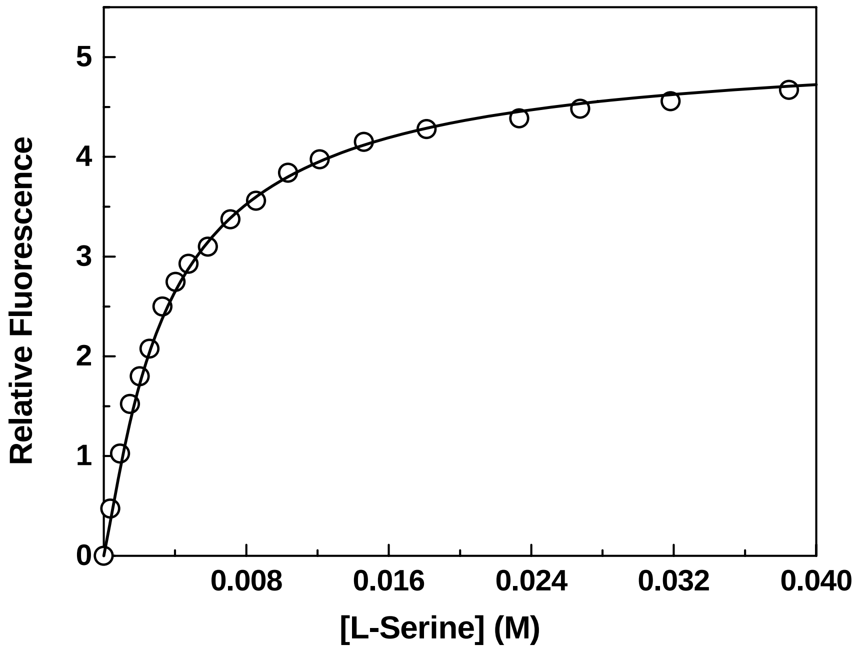

Titration of L-serine to StOASS at pH 9.5 (20 mM Tris, 20 mM NaCl). The concentration of proteins is 1.4 μM and 70 mM stock of L-serine is used for titration. The relative fluorescence is obtained as described in the materials and methods. Analysis of the binding data using eq (1) yields a value of equilibrium dissociation constant, Kd ~ 3.7 ± 0.1 mM.

Figure S4

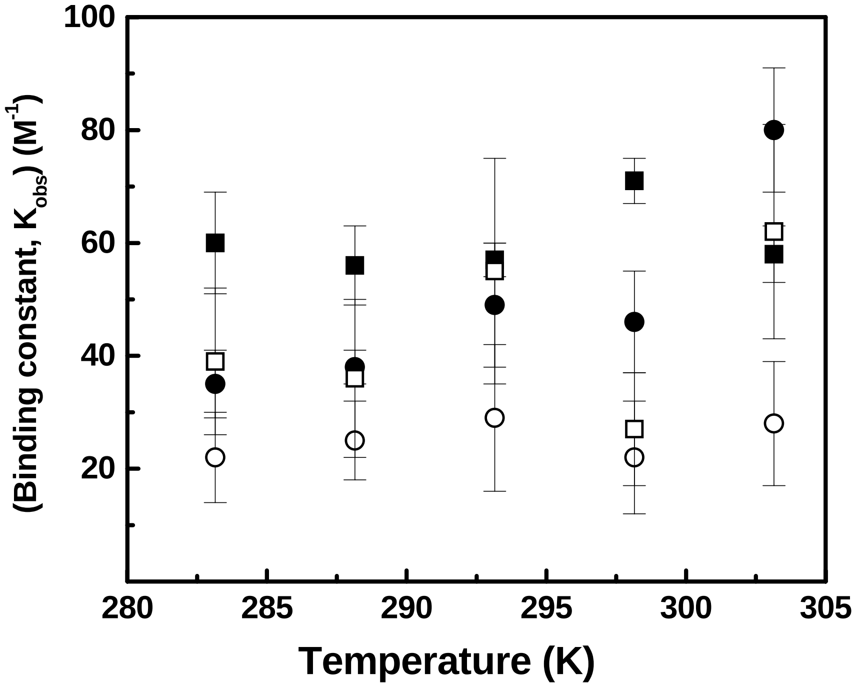

Temperature dependence of binding constants for cysteine and methionine binding to OASS; (■) *St*OASS-cysteine; (●) *St*OASS-methionine; (□) *Hi*OASS-cysteine; (○) *Hi*OASS-methionine.
