# Supplementary material for: Comparative thermodynamic studies on substrate and product binding of O-Acetylserine Sulfhydrylase reveals two different ligand recognition modes†
Source: BMC Biochem. 2011 Jun 2;12:31. doi: 10.1186/1471-2091-12-31 (PMC3141655; doi:10.1186/1471-2091-12-31)
Supplement: Additional file 2 — Spectroscopic and steady-state kinetic characterization of OASS. Catalytic competency of enzyme was analyzed by spectroscopic and kinetic experiments. [file 1471-2091-12-31-S2.PDF]

Figure S1

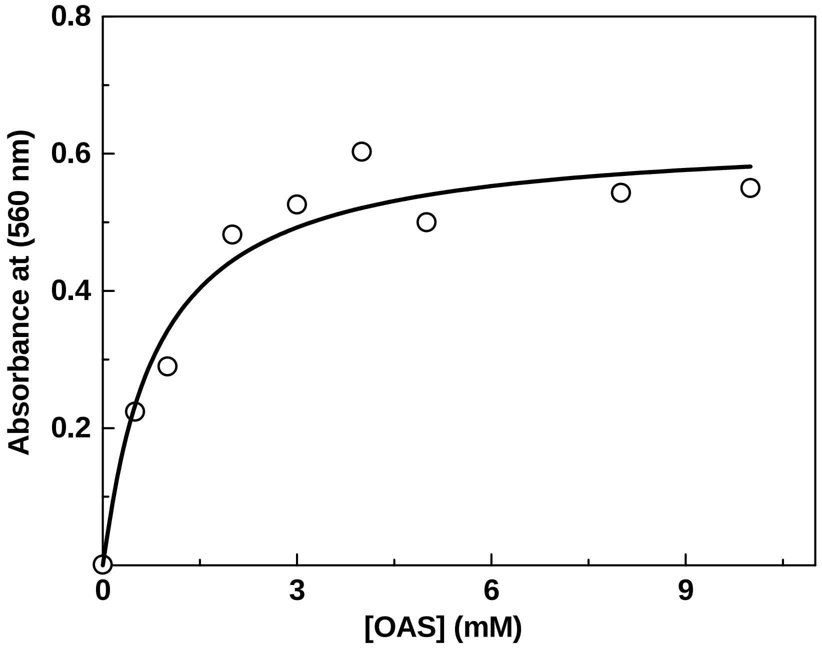

Steady state kinetic measurement of *StOASS* activity in the reaction buffer (as in Fig S2). OASS activity was measured by monitoring the cysteine formation. Data were fitted to Michaelis-Menten equation using origin 5.0. The K_m(OAS) was estimated to be 0.89 mM.

Enzyme assay: Assay is based on the specific reaction of ninhydrin with cysteine under strong acidic conditions and monitoring of modified cysteine (ninhydrin-cysteine adduct) at 560 nm. The reaction was allowed to proceed for 20 minutes (30 °C, linear phase) with varied concentrations of OAS (1- 10 mM, 2 mM Na_2S) in the reaction buffer (0.1M Hepes, pH 7.5, 150 µl reaction volume). The reaction was terminated with 5% TFA and centrifuged at 11000 rpm for 2 min. 125 µl glacial acetic acid + 125 µl acidic ninhydrin reagent were added to 125 µl of supernatant. Samples were boiled for 10 min at 100°C and cooled to room temperature. 600 µl of chilled absolute ethanol was added to the mix and the absorbance at 560 nm was used for determining the amount of cysteine present in the sample. Cysteine liberated was determined from a standard curve plotted by using known concentrations of cysteine.

Figure S2

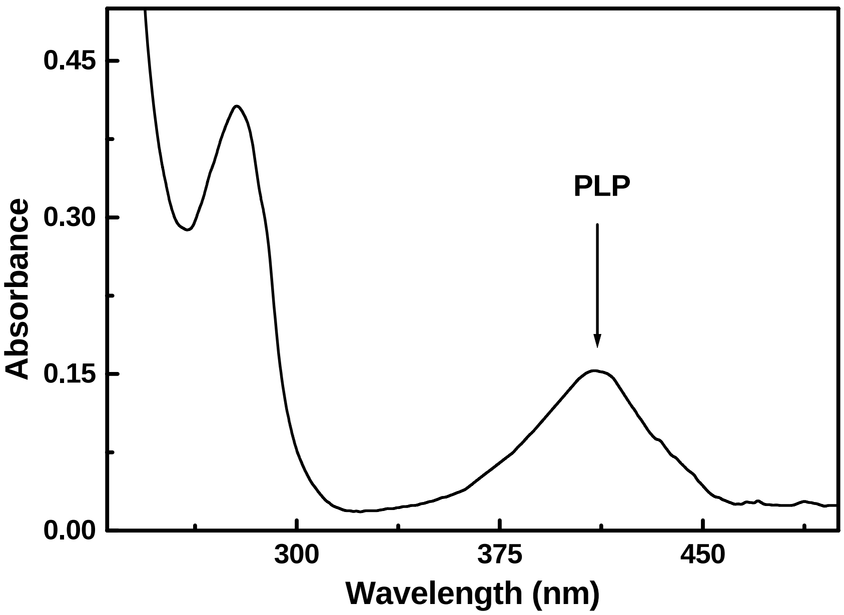

Absorbance spectrum of *StOASS* in the reaction buffer (25 mM HEPES, pH 7.8, 20 mM NaCl). Absorbance at 280 nm is 0.401 and at 412 is 0.152. The ratio of A280/A412 yields ~ 2.63. A ratio of ~ 3.4 or less is expected for the 1:1 stoichiometry of one PLP bound to single active site of OASS [24].

Figure S3

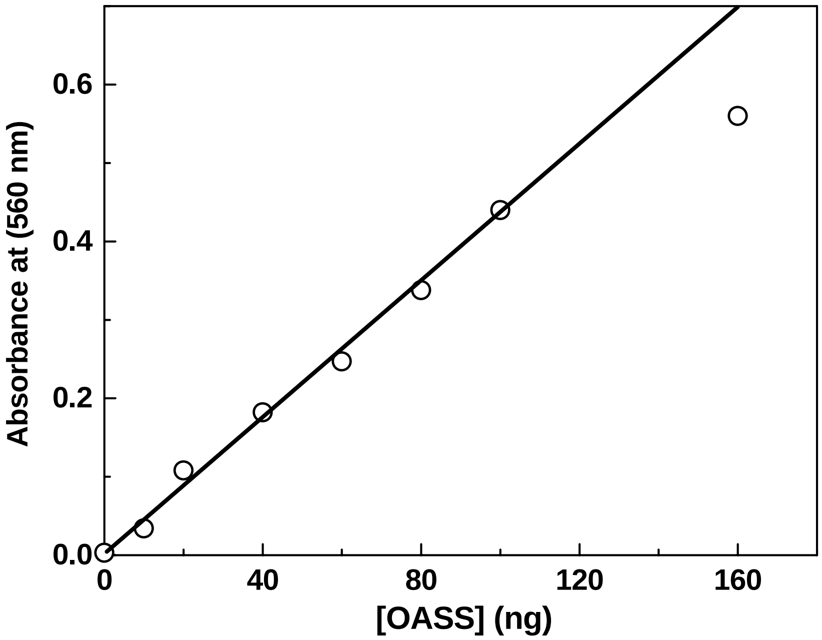

Determination of enzyme activity as a function of *St*OASS concentration at saturated substrate (4 mM OAS & 3 mM Na2S) concentration. Reactions were performed in HEPES buffer (25 mM, pH.7.8, 25 mM NaCl) and reaction was allowed to proceed for 20 minutes. The activity of enzyme increases linearly as amount of enzyme is increased. The expected zero order kinetics observed as a function of enzyme concentration indicates that our enzyme preparations are active.
